# The Force Awakening in HbA1c Control: A Systematic Review and Meta-Analysis on the Efficacy of High-Intensity and Endurance Exercise in Patients With Type 2 Diabetes Mellitus

**DOI:** 10.7759/cureus.73401

**Published:** 2024-11-10

**Authors:** Felipe A Muñoz Rossi, Jose A Cabarcas Rua, Néstor Israel Quinapanta Castro, Sara I Cedillo Orellana, Melissa Báez, Jonathan Coronel, Diana Marcela Zambrano Delgado, Vanessa Mejia Nates, Priscila A Leon, Antonio J Reche Martinez

**Affiliations:** 1 Internal Medicine, National University of Colombia, Bogota, COL; 2 Internal Medicine, University of Buenos Aires, Buenos Aires, ARG; 3 Research and Biostatistics, Autonomous Regional University of the Andes, Ambato, ECU; 4 Dentistry, Universidad Católica de Cuenca, Cuenca, ECU; 5 Medicine, Pontifical Catholic University of Ecuador, Quito, ECU; 6 Obstetrics and Gynecology, Hospital Materno Infantil José Domingo de Obaldía, David, PAN; 7 Surgery, Pereira University of Technology, Pasto, COL; 8 Emergency, Rey David Clinic, Cali, COL; 9 Endodontics, Universidad Católica de Cuenca, Cuenca, ECU; 10 Laboratory Medicine, Puerto Real University Hospital, Puerto Real, ESP

**Keywords:** aerobic exercise, diabetes mellitus type 2, glycated hemoglobin, high-intensity interval training (hiit), resistance training

## Abstract

The increase in the global prevalence of type 2 diabetes mellitus (DM2), driven mainly by obesity and physical inactivity, has increased interest in various nonpharmacological therapies. This systematic review aims to establish the effectiveness of high-intensity interval training (HIIT) and resistance exercise (RE) compared with continuous aerobic exercise in improving control in patients with DM2. We conducted a comprehensive search for clinical trials using databases such as MEDLINE (PubMed) and Web of Science. The search was performed using a controlled vocabulary (MeSH) together with Boolean operators, and the results were limited to English and Spanish. Secondary outcomes were improvements in VO_2_max and decreases in low-density lipoprotein (LDL). This study aims to explain evidence-based recommendations for primary care physicians on exercise therapies to improve glycemic management as well as cardiovascular health in people with DM2.

## Introduction and background

Type 2 diabetes mellitus (DM2) is a syndrome characterized by hyperglycemia, derived from various degrees of insulin resistance and insufficiency in production, acting in a combined manner [1].

Approximately 415 million individuals worldwide had diabetes in 2015, and DM2 is responsible for more than 90% of the cases. The number of people with DM2 is anticipated to grow by 10.2%, reaching 578 million in 2030 and 700 million in 2045 [2].

In Latin America, the prevalence of DM is between 8% and 13% in adults aged 20 to 75 [3]. In Argentina, a recent study reported that the prevalence of DM was 11.4% in the capital of Buenos Aires; however, in other regions, such as the northeast, it was 13.7%, the highest in the country [4].

Most instances of diabetes (90% to 95%) are attributable to DM2, where age and physical inactivity are key risk factors [5]. According to current guidelines for the treatment and management of DM2, the American College of Sports Medicine (ACSM) recommends that adults perform moderate-intensity cardiorespiratory exercise for a minimum of 30 minutes per day, at least five days per week, totaling at least 150 minutes per week [6]. Alternatively, vigorous-intensity cardiorespiratory exercise training is recommended for a minimum of 20 minutes per day, at least three days per week (equating to a minimum of 75 minutes per week), or a combination of moderate- and vigorous-intensity exercise to achieve a total energy expenditure of at least 500-1000 metabolic equivalent task (MET)/minute/week [7].

High-intensity interval training (HIIT) is a method that includes aerobic exercise at 65%-90% of maximum oxygen consumption (VO_2_max) or 75%-95% of maximum heart rate with active or passive recovery periods, which during the high-intensity phase can be between 10 seconds and four minutes in duration, while the recovery phase varies between 12 seconds and five minutes [7]. In turn, this strategy reduces the training time commitment while maximizing cardiometabolic adaptations, as well as allowing stability of glucose levels in people with DM, reducing total daily insulin requirements, and promoting improvements in the cardiometabolic risk profile [8].

Therefore, the high intensity and quick duration of HIIT exercise make it more appealing to patients with time restrictions or trouble completing longer, lower-intensity exercises. However, the level necessary may not be suitable for many patients [9].

Resistance exercise is defined as any sort of exercise that demands the contraction of muscles against a weight. This sort of exercise is generally conducted with the help of external weights, weight machines, or bodyweight exercises, with the objective of building muscular strength, size, and power [10].

The genetic and molecular mechanisms induced by resistance exercise (RE) are unique, as each exercise modality activates and represses specific genes and cell signaling pathways. Although the extent to which mitochondrial adaptations are related to RE has not been fully explored, uncertainty persists as to how RE can potentiate these mitochondrial modifications and provide benefits in patients with DM and obesity [9].

The goal of the intervention is to improve glycosylated hemoglobin (HbA1c) levels in people with DM2 by emphasizing physical activity through HIIT and RE, as opposed to continuous aerobic exercise. The effectiveness of this intervention may be shown by numerous techniques, such as enhanced insulin sensitivity. This development may lead to improved control of blood glucose levels and, hence, HbA1c values [11].

The importance of physical exercise is crucial for people with DM2 as well as for the general population. However, studies of this type of protocol continue to show varied outcomes; therefore, this review presents the latest significant scientific findings to determine whether alternative methods (HIIT-RE) can be as effective or superior to continuous aerobic exercise.

## Review

Methods

This systematic review will include clinical trials. Participants will be adults aged 18 to 65. Pregnant women, patients with physical limitations that hinder the effectiveness of the intervention, and patients with DM2 with chronic kidney disease stage III or higher will be excluded. Compared to continuous aerobic exercise, the therapies of interest include predetermined HIIT and resistance training.

Our primary outcomes will focus on HbA1c, a critical marker for assessing glycemic control in patients with DM2, as well as the variability in HbA1c results across different forms of exercise, such as HIIT and resistance training. Changes in insulin sensitivity and anthropometric characteristics will be considered secondary outcomes to acquire a better knowledge of the complete effect that exercise has on the health of individuals, as well as the reduction of cardiovascular risk.

We will implement a comprehensive search method that uses limited vocabulary based on medical subject headings (MeSH) and free-text words. This search approach will consider variation spellings, synonyms, acronyms, and truncation to discover research that is important to interest.

Field labels and Boolean operators will be used, with language restrictions to English and Spanish. The search formula will include terms such as "Diabetes Mellitus, Type 2" combined with "High-Intensity Interval Training", "Resistance Training", and "Exercise". We will search electronic databases such as PubMed MEDLINE and Web of Science, covering records through February 2024. Additionally, the reference lists of the studies that we have chosen will be examined.

Studies that have been discovered by the search technique will be independently reviewed by the authors, who will use the Rayyan platform to examine the titles, abstracts, and full texts of the studies. A consensus will be reached to moderate disagreements. Data extraction will be performed using a pilot form, which will collect information such as author, type of exercise, duration, number of participants, year, country, study design, route of administration, study date, scale used, diagnostic criteria, inclusion/exclusion criteria, participants' baseline information, comparator characteristics, and study registration number.

The Cochrane Risk of Bias Tool for Randomized Trials (RoB 2.0) will be used to provide an independent evaluation of the potential for bias in the included studies. The review will examine various topics, including the production of random sequences, the concealment of allocations, the blinding of participants and staff, inadequate outcome data, and selective reporting. The overall risk of bias will be rated according to Tramacere (2015) standards, including low, high, and uncertain risk categories. Disagreements shall be addressed by consensus or expert opinion.

Depending on the consistency of outcome measures across trials, the measures of treatment impact will include odds ratios (ORs) with confidence intervals (CIs) of 95% for dichotomous data and mean difference (MD) or standardized mean difference (SMD) for continuous data. These measurements will be used to determine the effectiveness of the therapy. The intervention groups for each trial will be specified in the table "Characteristics of included studies" and utilized for appropriate analysis. Any missing data will be remedied by getting in touch with the authors of the research when required.

Statistical heterogeneity will be examined using the I statistic, with a threshold of 50% indicating substantial heterogeneity. In such circumstances, a random-effects model will be employed. Information bias will be studied using funnel plots and Egger's test if at least 10 randomized controlled trials (RCTs) are included.

The data synthesis will comprise a narrative description of the findings for each outcome. The quality of evidence will be rated using the GRADE (Grading of Recommendations Assessment, Development, and Evaluation) approach, considering features such as risk of bias, inconsistency, indirectness, imprecision, and publication bias.

This systematic review with meta-analysis is filed in PROSPERO (Prospective International Register of Systematic Reviews) with the number CRD42024568207. The whole protocol is published in the PROSPERO database, covering the goals, inclusion and exclusion criteria, search tactics, and methods of analysis. This registry and methodology ensure the openness and reproducibility of our investigation.

Results

Search Results

**Figure 1:**
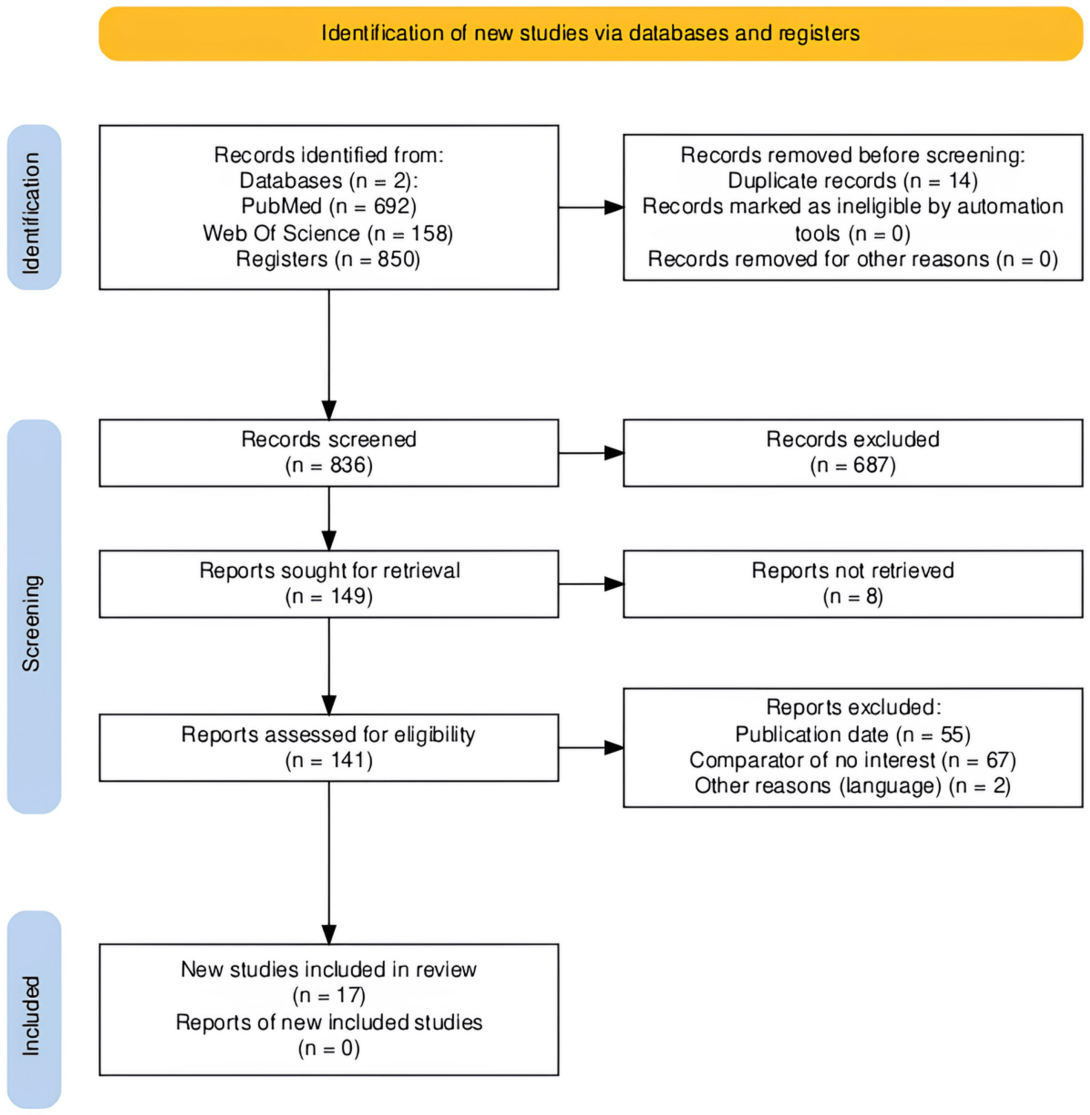
PRISMA flow diagram for systematic review PRISMA (Preferred Reporting Items for Systematic Reviews and Meta-Analyses)

In the present study, 850 references were identified from the electronic search of databases and the abovementioned sources. After elimination of duplicates (n = 14) and initial screening by title and abstract, 687 references were excluded, 149 references were eligible for full-text analysis; however, 71 were excluded for not corresponding to clinical trials, 55 studies for having full text and/or being more than 10 years old since it was written, 67 studies for a different comparator of interest, and finally two for not being available in full text in the English language. Therefore, 17 studies were included for analysis in this systematic review, as shown in the PRISMA (Preferred Reporting Items for Systematic Reviews and Meta-Analyses) flowchart (Figure 1).

Included Studies

Table 1 shows the characteristics of the included studies. With a total of 616 patients, the median age is 56.2 years, with a proportion of males 45.68% (n = 381) and females 41.12% (n = 343), respectively. There were 13 studies that assessed HbA1c decrease as part of their primary outcomes, while only eight studies assessed VO_2_max. Seventeen clinical trials were included for a total of 616 subjects with DM2. The studies were conducted in different regions: three studies in England, three in Canada, two in China, two in Denmark, one in Taiwan, one in Thailand, and others in Norway, India, Chile, Brazil, and the USA.

**Table 1:** Characteristics of the included clinical trials ↑ Increasing indicator; ↓ decreasing indicator; ↔ unchanged indicator AE (Aerobic Exercise); AMS (Accumulated Million Steps); BE (Burst Exercise); BMI (Body Mass Index); CG (Control Group); CON (Continuous Aerobic Training); DT2 (Diabetes Mellitus 2); END (Moderate-Intensity Endurance Training); GP (Glucosa Plasmática); HAIT (High Aerobic Intensity training); Hba1c (Hemoglobina Glicosilada); HI (High Intensity); HIIT (High-Intensity Interval Training); HVE (High-Volume Exercise); INT (Interval Aerobic Training); LDL (Low-Density Lipoprotein); LI (Low Intensity); LS (Lifestyle Intervention); MICT (Moderate-Intensity Continuous Exercise Training); MIT (Moderate- Intensity Training); MIW (Moderate-Intensity Walking); HRpeak (Peak Heart Rate); REHIT (Reduced-Exertion High-Intensity Training); RM (Repetition Maximum); RT (Resistance Training); SC (Standard Care); SED (Sedentary Control); SI (Insulin Sensitivity); T2D (Type 2 Diabetes); VO_2_Max ( Maximum Oxygen Uptake); WPeak (Peak Workload)

Author	Country	Population	Design	Intervention Time	Intervention	Primary Outcome	Secondary Outcomes
Petersen, et al. [8]	Denmark	48 T2D 15 Obese 15 Lean 18	HIIT Pre vs. Post T2D	3 times/week for 8 weeks	A HIIT combination of cycling and rowing	Hba1c↓ P< 0.001 VO_2_Max↑ P< 0.001	GP ↓ LDL ↓ BMI ↓
Støa, et al. [12]	Germany	38 T2D	HAIT vs. MIT	3 times/week for 12 weeks	HAIT 4x4 min intensity of 85 and 95 HRpeak	Hba1c ↓ P 0.02 VO_2_Max↑ P <0.01	LDL ↓ BMI ↓
Ruffino, et al. [13]	England	16 T2D	REHIT vs. MIW	3 times/week for 8 weeks	REHIT 10 min. Cycling at 25W interspersed with 1 or 2 Wingate cycling sessions	VO_2_Max↑ P < 0.05	LDL ↑ GP ↓
Lee, et al. [14]	Taiwan	120 T2D	AE vs. AMS vs. CG	5 times/week, 30 min for 1 year	AE 5 Times/Week 30 min	Hba1c ↓ P < 0.001	GP ↓
Mitranium, et al. [15]	Thailand	43 T2D	INT vs. CON vs. SED	3 times/week for 12 weeks	INT. Warm-up 50% VO_2_Peak—maintaining that intensity for 20min and 5min cooldown with 2 progressive increases	Hba1c ↓ P < 0.05	LDL ↓ BMI ↓
Johansen, et al. [16]	Denmark	98 T2D	LS vs. SC.	5-6 Times/Week for 1 year	LS 5-6 AE sessions, and 2 to 3 sessions combined with RT	Hba1c ↓ P 0.15 VO_2_Max ↑ P < 0.01	BMI↓
Yang, et al. [17]	Canada	51 T2D	AE RT1 (Usual care) LI (15RM) high (15) vs. RT2 HI (7RM) low (7) RT3 LI (15RM) high (15)	5 times/week for 6 months	RT2 3 sets of 7 repetitions at approx. 75% of their 1-RM for hypertrophic stimulus	Hba1c ↑ P < 0.001	BMI ↔
Cassidy, et al. [18]	United Kingdom	23 T2D	HIIT vs. SC	3 times/week for 12 weeks	HIIT 36 Ergometry Sessions: 5-min warm-up, followed by 5 intervals of pedaling cadence > 80 rpm/min, and a 3-min recovery cycle with a progressive increase	Hba1c ↓ P < 0.05	Liver Fat ↓
Pahra, et al. [19]	India	64 T2D	Group A vs. B	Daily, for 120 days	Group A Walking 15 min after meals from days 1–60. Then, a single exercise before breakfast, a moderate-intensity walk of 45 min before breakfast from days 61-120 Group B, was reversed.	Hba1c ↓ P < 0.001	GP ↓
Alvarez, et al. [20]	Chile	23 T2D	HIIT vs. CG	3 times/week for 16 weeks	HIIT 8 intervals of jogging/running for 30 seconds interspersed with 120 seconds of low-intensity walking with a progressive increase in jogging/walking and a decrease in walking.	LDL ↓	BMI ↓
Pandey, et al. [21]	Canada	40 T2D	BE vs. MICT	3 times/day for 5 days for 12 weeks	BE continuous exercise of 10 min high-intensity burst exercise (85% HRmax) 1-min warm-up and cool-down	Hba1c ↓ P < 0.001	LDL ↓ BMI ↓
Li, et al. [22]	China	54 T2D	RT vs. AE	5 times/week for 12 weeks	RT -warm-up 5min Squat with a resistance band and neck flexion and extension with a resistance band. 15-20 repetitions for women, and 20-25 for men. 3 sets of movement intervals of 2-3 min between sets	Hba1c ↓	LDL ↓ BMI ↓
Cassidy, et al. [23]	United Kingdom	22 T2D	HIIT vs. SC	3 times/week for 12 weeks	HIIT: 36 cycling sessions: warm-up (5 min), then 5 intervals of pedaling cadence greater than 80 RPM interspersed with a 3 min recovery period of 90 passive 60 resistance to the band for the upper body, and 30-sec preparations for the next work.	Hba1c ↓ P 0.151 VO_2_Max ↑ P 0.57	BMI↓
Madsen, et al. [24]	Denmark	23 13 Healthy 10 T2D	HIIT	3 times/week for 8 weeks	Warm-up 5 min, then intervals of 10x1 min interspersed with 1 min recovery Cadence 70 RPM, 5 min cooldown, 30 min per session	VO_2_Max ↑	LDL ↓ BMI ↓
MacDonald, et al. [25]	United Kingdom	92 T2D	U-TURN vs. CG	240 min/week for 4 months, then at least 300 min/week for 8 months	HVE 240 min/week of AE and RT (4M), then at least 300 min/week for the rest of the 8 months.	Hba1c ↑ VO_2_Max ↑	LDL ↑ BMI ↔
Winding, et al. [26]	Denmark	32 T2D	HIIT vs. END	3 times/week for 11 weeks	END cycling 40min/session 50% Wpeak HIIT 20min/session 95% Wpeak	Hba1c ↓ P < 0.05	LDL↓ BMI↓
Li, et al. [27]	China	37 T2D	HIIT vs. MICT	5 times/week for 12 weeks	MICT 30min/session HRpeak oxygen consumption 50-70% HIIT 15min/session HRpeak oxygen consumption 80-95%	Hba1c↓ P 0.009 VO_2_Max ↑ P 0.001	BMI ↔

Primary and Secondary Outcomes

Reduction of glycosylated hemoglobin was evaluated in most of the included studies. However, in the study by Ruffino et al. [13], HbA1c was not considered an outcome assessed. MacDonald et al. [25] analyzed differences in HbA1c levels but focused on the delta of differences. The study by Madsen et al. [24] did not have the exact value of HbA1c, while in the study by Pahra et al. [19], the randomized interventions prevented an accurate assessment of this marker.

In total, there were 278 individuals in the experimental group and 246 individuals in the control group across the 13 studies included in the meta-analysis [8,12,14-18,20-23,26,27]. In individuals with DM2, HIIT and endurance exercises resulted in a significant decrease in HbA1c levels, evidenced by an estimated SMD of -0.45 (95% CI: -0.63, -0.28). According to the common-effects model, this difference is significant.

**Figure 2:**
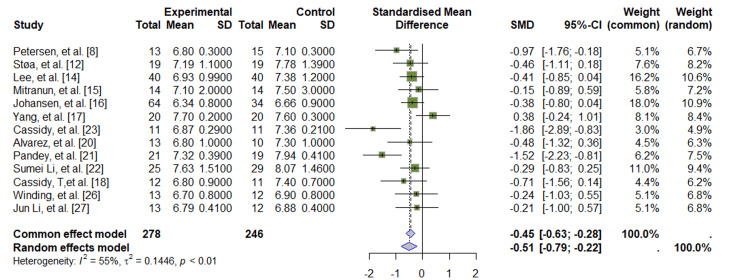
Forest plot: HbA1c (glycosylated hemoglobin) SD (Standard Deviation); SMD (Standardized Mean Difference); CI (Confidence Interval)

However, considering a heterogeneity between studies that was moderate (I^2^ = 55%), a random-effects model for MDs is performed to show if there is any variability between the results, with a similar effect of -0.51 (95% CI: -0.79; -0.22), confirming our alternative hypothesis. Even though there is some variation in the results of the studies mentioned above, this is not enough to disprove the overall results shown in the forest plot (Figure 2).

Maximal Oxygen Consumption (VO_^2^_max)

**Figure 3:**
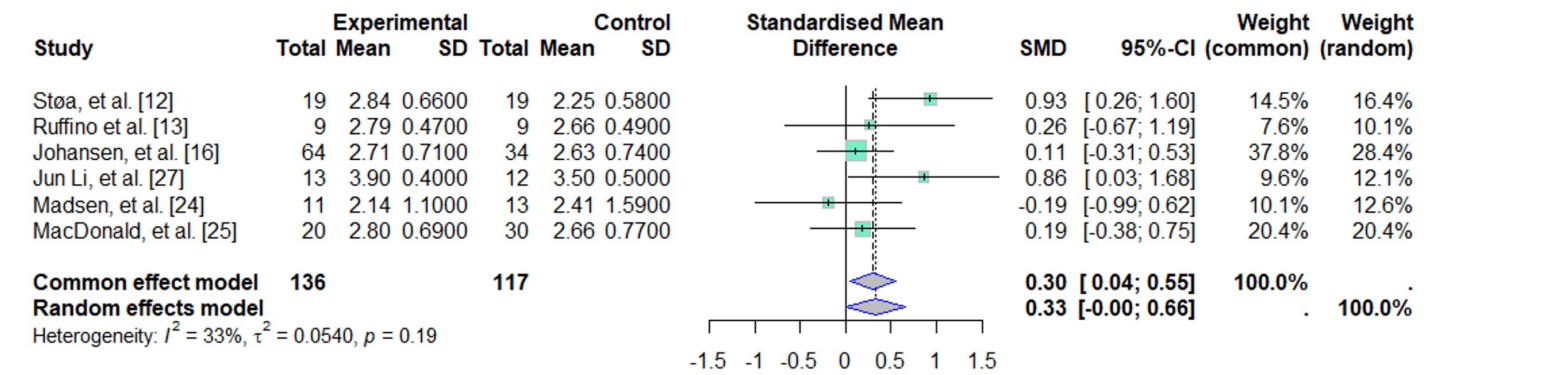
Forest plot: VO2max SD (Standard Deviation); SMD (Standardized Mean Difference); CI (Confidence Interval)

Six studies were included, with a total of 136 subjects in the experimental group and 117 in the control group [12,13,16,24,25,27]. Regarding the combined effect, for the common-effects model, the MDs evidenced a positive effect in the increase of oxygen consumption of 0.30 (95%CI: 0.04-0.55) compared to the control group; however, there is moderate heterogeneity between studies (I^2^ = 33%), but with a controversial finding because the CI includes the null value (Figure 3).

Low-Density Lipoprotein (LDL) Levels

**Figure 4:**
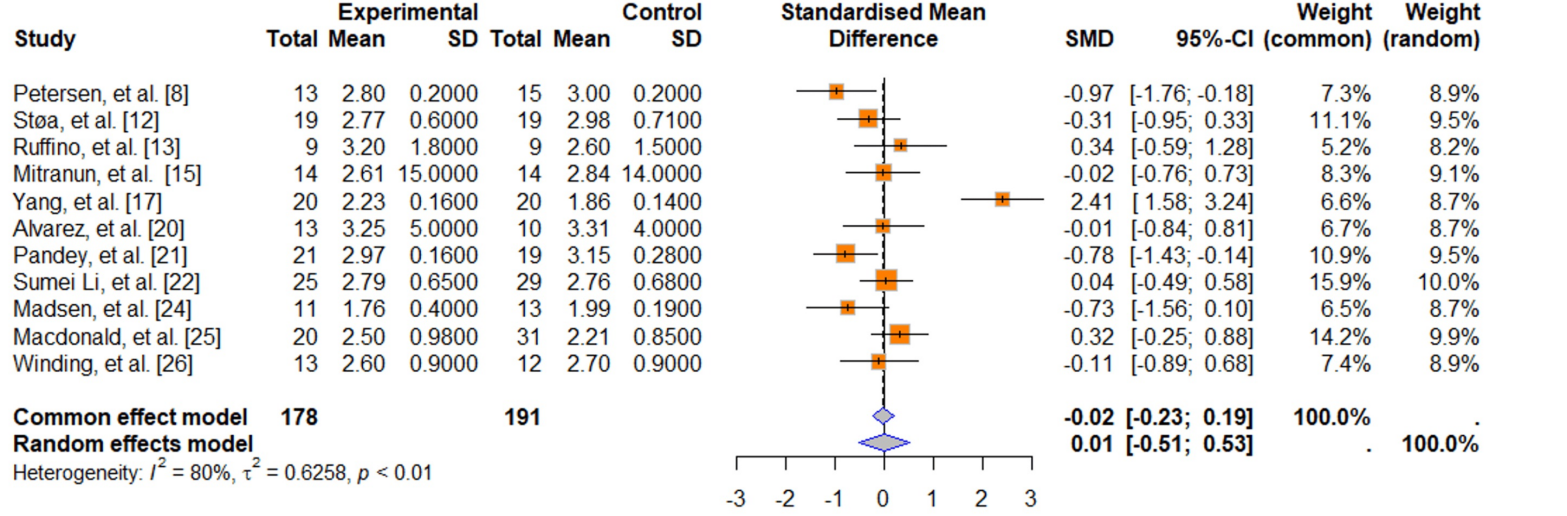
Forest plot: LDL levels SD (Standard Deviation); SMD (Standardized Mean Difference); CI (Confidence Interval); LDL (Low-Density Lipoprotein)

Concerning this item, 11 articles were included, with a total of 178 subjects in the experimental arm and 191 in the control arm. Only Petersen et al. [8] and Pandey et al. [21] found statistically significant differences in LDL reduction; the pooled estimate of the MD suggests that there is no significant difference in LDL levels, with an SMD statistic of -0.02 (95%CI = -0.23; -0.19). The heterogeneity between studies is high, with a p-value of 0.01, indicating that there is statistically significant evidence of heterogeneity (Figure 4).

Risks of Bias of the Included Studies

In this systematic review of selected studies, a rigorous assessment of the risks of bias associated with each paper was conducted using the ROB 2.0 tool. The assessment was conducted based on a review of five key dimensions: D1: the randomization process; D2: the deviations from the intended interventions; D3: the missing outcome data; D4: the measurement of the outcome; and D5: the selective reporting of results.

Regarding biases, 10 studies presented a moderate risk of bias, six had a high risk, all mainly in the randomization process, with a high risk of selection bias; none presented a low risk.

**Figure 5:**
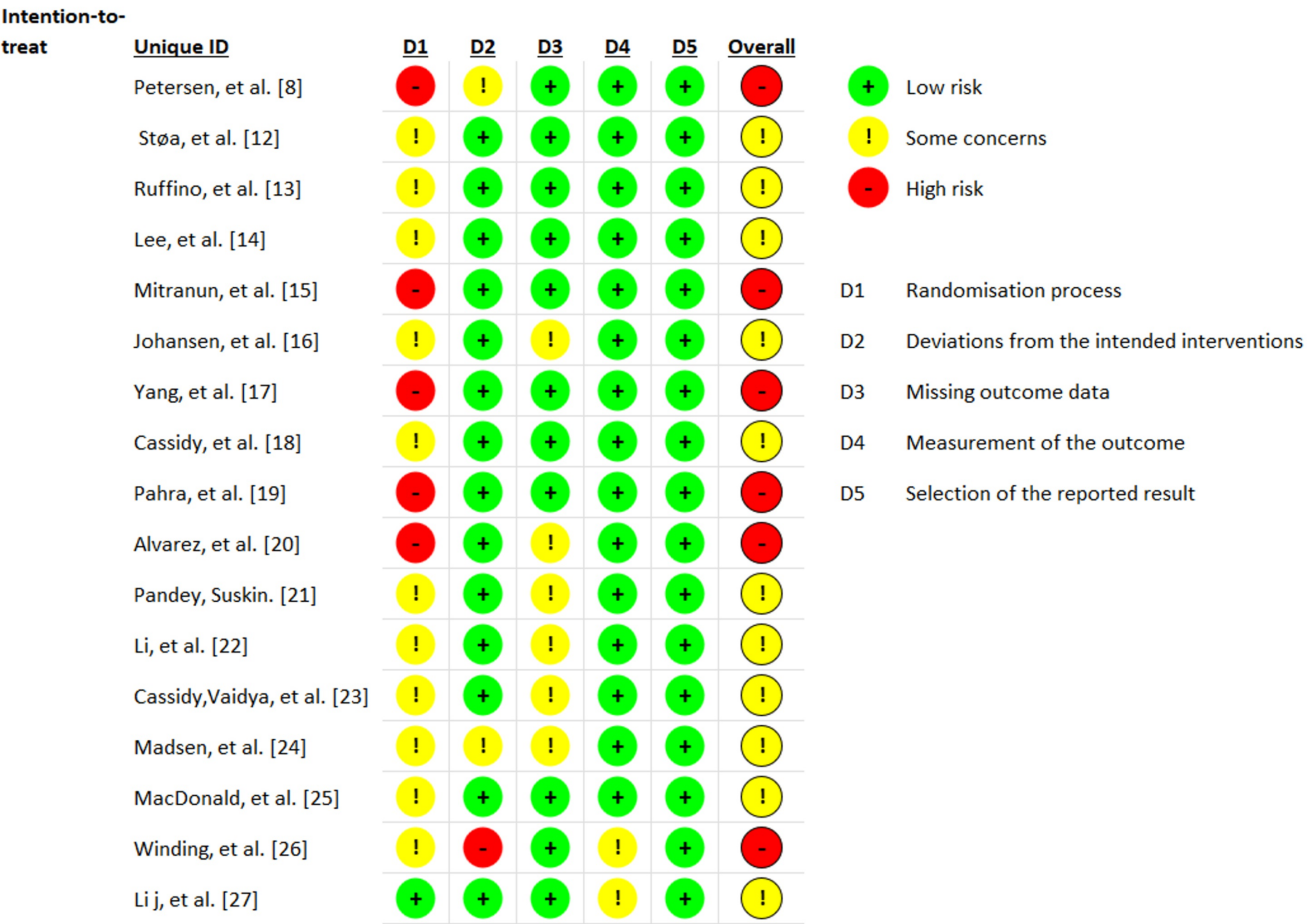
Summary of the risk of bias (RoB 2.0)

**Figure 6:**
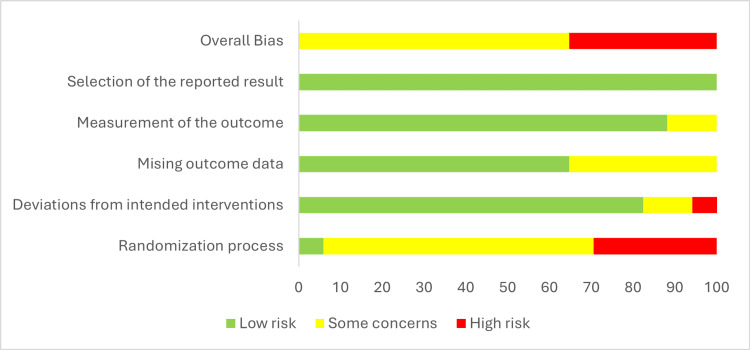
Risk of bias: each factor is presented as percentages overall among the included studies

Some studies [8,15,17,19-21] presented clinical differences in baseline characteristics between groups, which would lead to an important selection bias. Only the study by Lee et al. explicitly presented an intention-to-treat analysis in the report [14]. These findings highlight serious design problems in subjecting these results to external validity. The results of the risk of bias assessment are presented in Figure 5, and Figure 6 presents each factor globally for the included studies.

Evaluation of publication bias

Publication Bias for HbA1c

**Figure 7:**
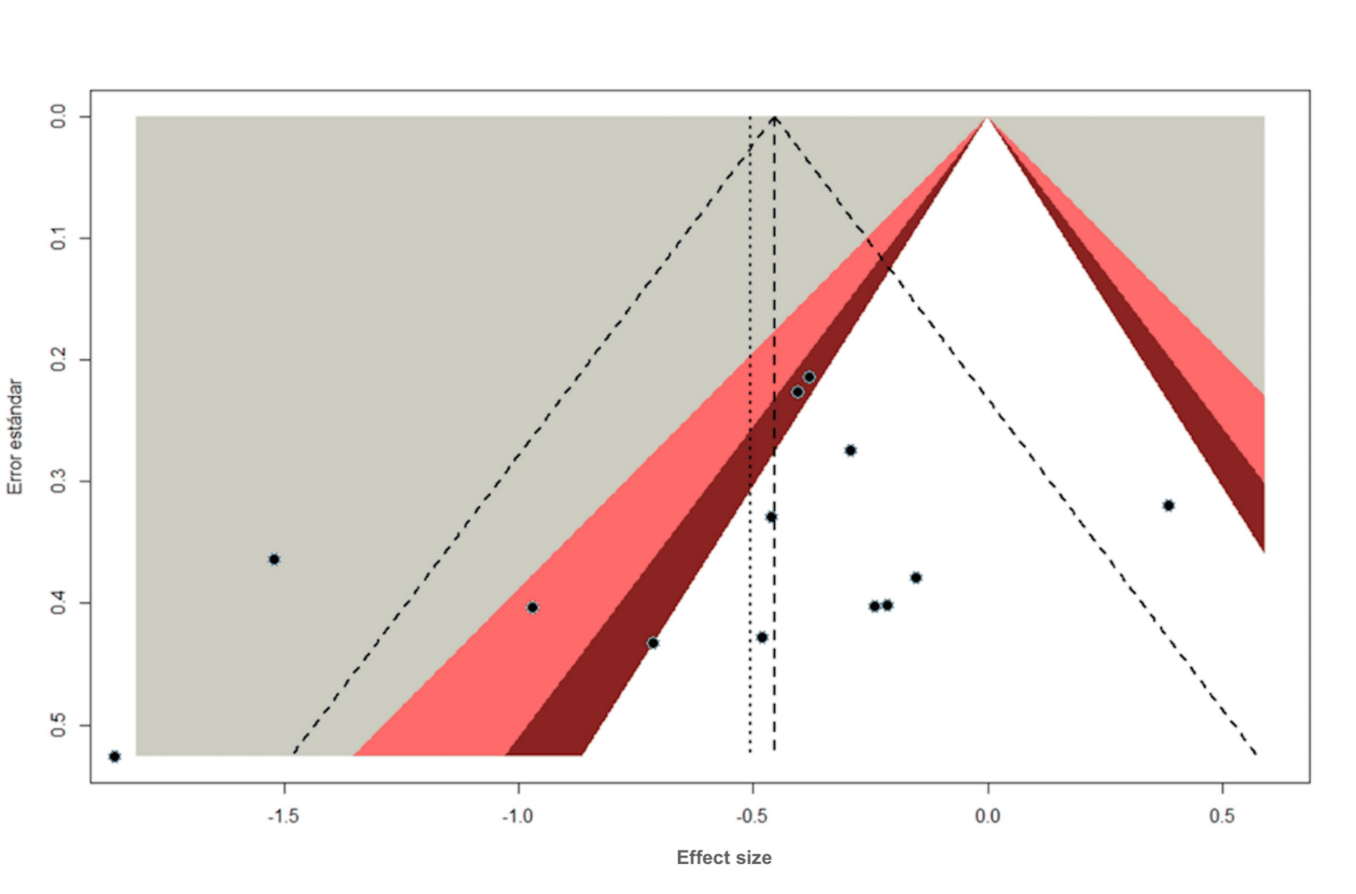
Funnel plot: HbA1c

Regarding the publication bias observed in the results obtained for HbA1c, they have a symmetrical distribution. However, there is a dispersion of certain points on the left side of the funnel plot, which may indicate a possibility of publication bias (Figure 7). For this, Egger's test is performed with an intercept of 0.1994 (standard error = 0.4967), which indicates the probability of publication bias. However, the test is performed with a p-value of 0.19, which is not statistically significant. As a result, we can conclude that there is insufficient evidence to support the presence of a significant publication bias on the HbA1c results that were found.

Publication Bias for LDL

**Figure 8:**
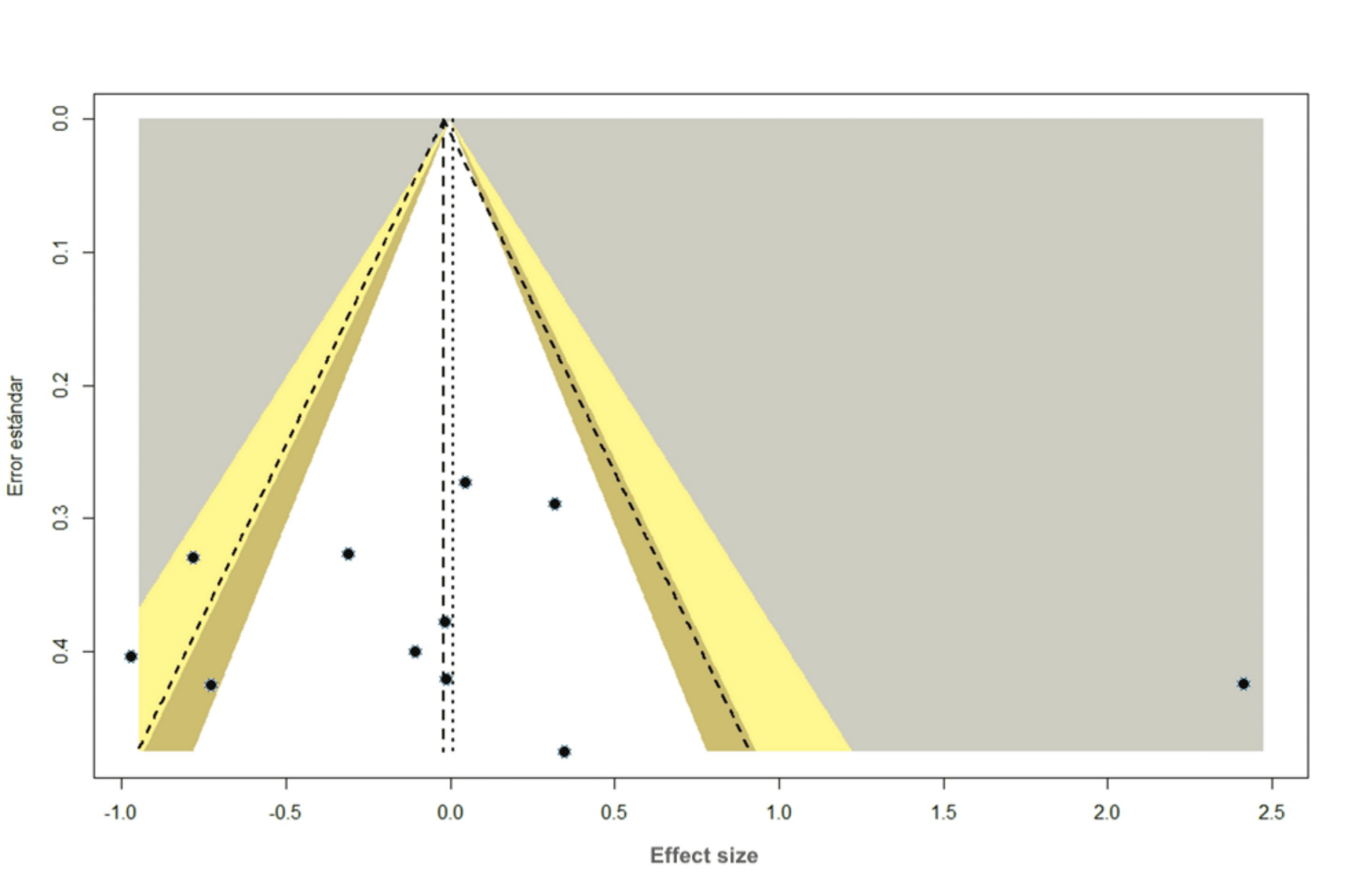
Funnel plot: LDL LDL (Low-Density Lipoprotein)

Most studies are at the bottom of the funnel plot, with a standard error greater than 0.2; however, with little asymmetry between studies, using Egger's statistic (t = -0.35, df = 9, p = 0.7348), no significant asymmetry was detected, suggesting no publication bias (Figure 8).

Discussion

The findings of the present investigation indicate that HIIT exercise and resistance exercise are undoubtedly effective interventions to improve HbA1c levels; however, with more modest effects on VO_2_max and LDL levels in patients with DM2, the 17 clinical trials selected for the meta-analysis were conducted in different regions, which gives a global perspective of the results found.

Therefore, it is noteworthy that HIIT and resistance exercise have a moderate effect on the reduction of HbA1c levels; however, this effect varies considerably between studies, given by a moderate-high heterogeneity (I^2^ = 55%). Nonetheless, measures such as the random-effects model were used to confirm the positive effects found in this research; therefore, we consider that interventions based on HIIT and RE are effective measures to reduce HbA1c levels in patients with DM2.

Although we did not find similar systematic reviews evaluating both HIIT and RE, we found meta-analyses evaluating them separately. Strasser et al. assessed the effect of RE on glucose intolerance under a meta-analysis including 13 RCTs and found that RE reduced HbA1c by 0.48% (95% CI = -0.76; -0.21), findings similar to those found in our study [28].

Additionally, a meta-analysis demonstrated that aerobic and RE can be effective in glycemic control in patients with DM2; however, combined exercise of these two modalities was found to be more beneficial [29]. These results, therefore, complement our research and suggest that a combination of different types of exercise may further enhance the observed benefits.

In addition, the VO_2_max analysis of the six studies revealed a positive effect on the increase in oxygen consumption. However, our study could not demonstrate that these differences were statistically significant; similar findings were found in a systematic review with inconclusive results (p = 0.21) with respect to VO_2_max [30].

On the other hand, LDL levels were evaluated in 11 studies, with a total of 178 subjects in the experimental group and 191 in the control group. A pooled estimate for the MD failing to demonstrate a significant reduction, coupled with high heterogeneity (p = 0.01), suggests that the data presented in our research are limited in their ability to be generalized.

Limitations

Despite the relevant results, we also highlight some limitations that should be considered when interpreting the results. First, we emphasize that the heterogeneity found in the analyses across studies for the different parameters is probably due to differences in the clinical trial designs and the various ways of measuring outcomes. Nevertheless, we used the random-effects model to address this heterogeneity, and the results should be interpreted with this caveat.

On the other hand, another limitation is the intrinsic risk of each of the included studies. The evaluation under the ROB 2.0 tool showed that 11 studies presented some considerations to be considered for the risk of bias, and six studies presented a high risk of bias, mainly in the randomization processes and in the selection bias. This placed the internal validity of the results in uncertainty and limited the ability to generalize the findings to diverse populations, with relevant repercussions on the external validity of the results. Furthermore, the lack of intention-to-treat analysis in the other included clinical trials highlights concerns about the reported data, except for one [14].

Furthermore, there are differences in the variability of the interventions and measures used in the studies. Some studies not only used HIIT and other REs but also different time intervals for the interventions of these modalities, which compromises the comparability of the reported data and has important implications for the observed heterogeneity.

Although several tests for publication bias were performed and no significant evidence of asymmetry was found, it is important to highlight the language bias because this review focused on clinical trials published in the English language. Because of this, the possibility of publication bias cannot be completely ruled out.

Finally, we consider that although studies from various regions were included, most were conducted in developed countries; therefore, their applicability at the local level in less developed countries, where resources and health conditions may differ considerably, is limited.

## Conclusions

Strength training and HIIT effectively lower HbA1c levels and enhance VO_2_max, stressing the necessity for organized programs that include both treatments as supplementary methods in the management of DM2. We suggest healthcare facilities tailor exercise advice for patients to optimize therapeutic effects. A future study should concentrate on the influence of physical exercise on particular subgroups of DM2 patients, including those with comorbidities and age extremes. Additionally, initiatives focused on boosting long-term adherence, motivation, and the utilization of exercise technology are critical to enhancing health outcomes in this group.
